# Green synthesized silver nanoparticles: Optimization, characterization, antimicrobial activity, and cytotoxicity study by hemolysis assay

**DOI:** 10.3389/fchem.2022.952006

**Published:** 2022-08-29

**Authors:** Nida Liaqat, Nazish Jahan, Khalil-ur-Rahman, Tauseef Anwar, Huma Qureshi

**Affiliations:** ^1^ Department of Chemistry, University of Agriculture, Faisalabad, Pakistan; ^2^ Department of Chemistry, Rawalpindi Women University, Rawalpindi, Pakistan; ^3^ Department of Biochemistry, University of Agriculture, Faisalabad, Pakistan; ^4^ Department of Botany, The Islamia University of Bahawalpur, Bahawalpur, Pakistan; ^5^ Department of Botany, University of Chakwal, Chakwal, Pakistan

**Keywords:** silver nanoparticles, green nanotechnology, zeta potential, dynamic light scattering, UV-visible spectrophotometer, antibacterial activity

## Abstract

Green nanotechnology has emerged as a viable option for the production of nanoparticles. The purpose of the current investigation was to synthesize silver nanoparticles (AgNPs) using *Eucalyptus camaldulensis* and *Terminalia arjuna* extracts, as well as their combinations, as green reducing and capping agents. The parameters (concentration of silver nitrate solution and plant extract, time, pH, and temperature) were optimized for maximal yields, regulated size, and stability of silver nanoparticles. The ultraviolet–visible spectrophotometer (UV-Vis) and the surface plasmon resonance band (SPR) were used to validate the synthesis of AgNPs. The size, shape, and stability of nanoparticles were assessed using a zeta analyzer and a scanning electron microscope (SEM). The biomolecules responsible for the reduction of silver ion (Ag^+^) and the stability of silver nanoparticles generated with the plant extracts were identified using Fourier-transform infrared spectroscopy (FTIR). The agar-well diffusion method was used to test the antimicrobial activity of biosynthesized nanoparticles against *Bacillus subtilis, Staphylococcus aureus*, *Pasteurella multocida*, and *Escherichia coli*. When 1 mM of silver nitrate (AgNO_3_) was added to plant extracts and incubated for 60 min at 75°C in a neutral medium, maximum nanoparticles were produced. Biosynthesized silver nanoparticles were stable, spherical, and monodispersed according to zeta potential and scanning electron microscopy. Silver nanoparticles synthesized with combination 2 and *T. arjuna* showed the highest zone of inhibition (16 mm) against *B. subtilis* while combination 3 showed the largest zone of inhibition against *S. aureus* (17 ± 0.8)*.* It was concluded that greenly produced silver nanoparticles showed good antibacterial activity while causing negligible cytotoxicity.

## 1 Introduction

Metal nanoparticles have received much attention due to their remarkable physicochemical and optoelectronic properties in a wide range of applications (Yaqoob et al., 2020). Nano-silver (AgNPs) is a type of metallic nanoparticle with unique features and uses, particularly in the field of nanomedicine (Xu et al., 2020). Due to their distinct antibacterial qualities, silver nanoparticles have attracted the most attention among all other nanoparticles. Green synthesis is a new alternative method that was inspired by concerns about the synthesis of these materials, such as the use of harmful solvents and precursor chemicals and the production of toxic byproducts. This environmentally friendly method uses microbial, plant, or biological agents as reducing and capping agents. Green chemistry-produced silver nanoparticles present a novel and potential substitute for chemically-produced nanoparticles (Roy et al., 2019). Because of its easy, efficient, eco-friendly, and non-toxic approach, green nanotechnology (using enzymes, microorganisms, and plant/plant extract) is a potential alternative to pure nanotechnology (Zhang et al., 2020). Plants are made up of a variety of bioactive substances that can help with the production of metal nanoparticles by acting as reducing and stabilizing agents (Sharma et al., 2019; Begum et al., 2022). Bio-inspired syntheses result in nanoparticles of diverse forms and sizes depending on the synthesis process parameters (Khan et al., 2017). The synthesis conditions must be improved by using local plants.

Since there is no risk of bacterial or chemical contamination, there is less energy consumption with broader consequences, and it is simpler and easier to use plant materials for AgNP production than bacterial and chemical techniques. AgNPs’ biosynthesis employing plant extracts and microorganisms as reducing agents, as well as their antibacterial efficacy, have recently received a lot of attention. By bio-reducing silver nitrate with *Hagenia abyssinica* plant leaf extract, Melkamu and Bitew (2021) were able to create silver nanoparticles in a sustainable manner. *Streptococcus pneumoniae, Salmonella typhimurium*, and *Klebsiella pneumoniae* were all susceptible to the antibacterial action of the produced AgNPs. From the leaves of *Eugenia roxburghii*, silver nanoparticles were produced that efficiently prevented the growth of bacterial colonies that produce biofilm (Giri et al., 2022). Similar to this, AgNPs mediated by *Acorus calamus* (rhizome) extract have demonstrated excellent activity in a dose-dependent manner against *Staphylococcus aureus*, *Salmonella enterica*, *B. cereus*, *S. enterica*, and *E. coli* as well as exhibiting good stability and could be used as a potential antibacterial agent for a commercial application (Sudhakar et al., 2015). The bacterial strains *S. aureus* and *Pseudomonas aeruginosa* were inhibited by AgNPs produced using aqueous *Allium sativum* extract (Rastogi and Arunachalam, 2011). Activity against *P. aeruginosa, E. coli, S. aureus*, and *Bacillus subtilis* was demonstrated by *Acalypha indica* aqueous extract-mediated AgNPs. Flavonoids found in the petals of *Linium casablanca* showed excellent stability, antimicrobial action, and catalytic activity during the production of AgNPs. *E. coli* and *Salmonella* were more resistant to AgNPs’ antibacterial effects, while *Bacillus subtilis* and *S. aureus* were less (Luo et al., 2018). The MDR *Salmonella enterica* bacteria were resistant to ciprofloxacin, tetracycline, and cefotaxime. The green-mediated production of AgNPs utilizing *Myristica fragrans* dried seed extract has shown good bactericidal action against these bacteria (Balakrishnan et al., 2017). AgNPs employing *Boerhaavia diffusa* were tested against *Pseudomonas fluorescens, Aeromonas hydrophila*, and *Flavobacterium branchiophylum*, which are fish pathogens. In comparison to other test bacterial pathogens, AgNPs have shown great potential antibacterial efficacy against *F. branchiophylum* bacteria (Kumar et al., 2014). *E. coli, P. aeruginosa*, and *S. aureus* are human pathogens that can be effectively combatted by AgNPs made by *Skimmia laureola*. The silver was reduced and stabilized by capping with the phytochemicals found in the *S. laureola* extract, resulting in AgNPs that were spherical and hexagonal in shape and crystallized (Ahmed et al., 2015). *Aloe vera* (leaf)-derived AgNPs may have antimicrobial properties. The octahedron shape of the AgNPs showed greater antibacterial capabilities against *Bacillus cereus, S. aureus, Micrococcus luteus, E. coli*, and *K. pneumoniae*. The flavonoids and terpenoids present in the extract were responsible for the stability of the AgNPs (Logaranjan et al., 2016). The Gram-positive alpha and beta-hemolytic *Streptococcus* sp., *Streptococcus aureus, S. haemolyticus*, and *Bacillus* sp., as well as the Gram-negative bacteria *E. coli, E. faecalis, Proteus mirabilis, K. pneumonia*, and *P. aeruginosa*, were all susceptible to AgNPs made using the aqueous extract.

Plant extract biomolecules such as flavonoids, ketones, aldehydes, tannins, carboxylic acids, phenolics, and proteins are in charge of converting Ag^+^ to Ag^0^ in order to produce AgNPs. The biosynthesized AgNPs have many morphologies, diameters, and forms. Under various experimental settings, such as temperature, pH, the kinetics of interaction between metal salts and reducing agents, nature, and adsorption of capping agents, all these properties are significantly altered. AgNPs’ morphology, size, and shape all affect how they perform. The most critical area of research in nanoparticle synthesis is currently developing a method that can regulate the size, shape, stability, and physicochemical qualities. Recently, green metal nanoparticle production employing a variety of plants and plant products has been successfully completed (Rahuman et al., 2022).

*Terminalia arjuna* is known for its antibacterial, antiviral, cardioprotective, hypolipidemic, and antithrombotic properties (Thakur et al., 2021). It was chosen for the production of silver nanoparticles. Traditional medicine uses diverse parts such as bark, leaves, and fruits to treat a variety of diseases. The bark powder has been discovered to have a wide range of therapeutic qualities. Various phytoconstituents including arjunin, arjunic acid, qudranoside VIII, terminarjunoside I and II, luteolin, baicalein, kempferol, oligomeric proanthocyanidins, pelargonidin, quercetin, β-sitosterol, hentriacontane, methyl oleaolate, and myristyl oleate are obtained from stem bark, root, and fruits of *T. arjuna* (Khatri et al., 2017). *Eucalyptus camaldulensis*, on the other hand, has been utilized as an antibacterial agent in a variety of illnesses (Shagal et al., 2012). Several investigations have shown that *Eucalyptus* species leaf extracts have antibacterial effects against a variety of microbes. The phytochemical components found in *E. camaldulensis* leaf extracts, including flavonoids, alkaloids, pigments, terpenes, phenolics, starches, steroids, and essential oils, are known to have antibacterial action (Ceyhan et al., 2012). Essential oils (aromandendrene myrtenal, borneol, camphene, carvacrol citronellal citronellyl acetate, and cryptone-α-terpenyl acetate), flavonoids (apigenin, chrysin, flavone, luteolin, eriodictyol, hesperetin, naringenin, and pinocembrin), triterpenoids (oleanolic acid, maslinic acid, camaldulic acid, and camaldulensic acid) are the main constituents present in *E. camaldulensis* (Nagpal et al., 2010; Shah et al., 2016). The current study is aimed at the optimization of physical parameters for the synthesis of AgNPs using *T. arjuna* bark and *E. camaldulensis* leaf extracts. These plants were chosen because they are widely used in folk medicine and are readily available locally. The optimized nanoparticles were also characterized and analyzed for antibacterial and hemolytic properties. Combined extracts were formulated from the mentioned aqueous extracts to investigate the synergistic effect of the two different extracts.

## 2 Materials and methods

The present research was designed for the green synthesis of silver nanoparticles from plant extracts of *Euclayptus cameldulensis*, *Terminalia arjuna,* and their combinations. The preparation and optimization of green synthesized silver nanoparticles have been carried out in the natural product laboratory of the Department of Chemistry, University of Agriculture, Faisalabad. Zeta potential and SEM analysis of green synthesized AgNPs were performed at National Institute for Biotechnology and Genetic Engineering (NIBGI) and National Textile University (NTU) Faisalabad. The departments provided analytical-grade chemicals that were employed directly without further purification.

### 2.1 Preparation of plant extracts and their combinations

The healthy leaves of *E. camaldulensis* and bark of *T. arjuna* were collected from the University of Agriculture in Faisalabad, Pakistan. The collected plant specimens were identified by using the Flora of Pakistan (http://www.tropicos.org/Project/Pakistan), and World Flora Online (http://www.worldfloraonline.org/) was followed to find the correct scientific name. The voucher specimens (*E. camaldulensis;* UAF-54 and *T. arjuna;* UAF-88) were deposited in the herbarium of the University of Agriculture, Faisalabad. The dried plant samples were ground into a powder, and then aqueous extracts of each sample were made by adding water at a 10:1 (w:v) ratio to the powder. The mixtures were immediately heated for 10 min at 60°C to deactivate the enzymes. The mixtures were filtered using Whatman filter paper (Grade no. 1), and the supernatants were saved for use in the production of AgNP. Then, 10 ml of plant extract and 90 ml of 1 mM silver nitrate (AgNO_3_) solution were mixed in a flask for the production of AgNP. A stable dark color emerged after 48 h of the reaction being stored at room temperature and in the dark. Following storage at 4°C, the combination was kept (Castillo-Henríquez et al., 2020; Aabed and Mohammed, 2021). The synergistic/ antagonistic effects of medicinal plant extracts based against microbes were also investigated. The combinations were formed by extracts of *E. camaldulensis* and *T. arjuna* in ratios of 1:1 (combination 1), 2:1 (combination 2), and 1:2 (combination 3).

### 2.2 Biosynthesis of silver nanoparticles

Plant extract (10 ml) was mixed with 90 ml of 1 mM silver nitrate (AgNO_3_) solution. The reaction mixture was incubated in the dark. Control was maintained without plant extracts. The individual plants and their combinations were used for the biosynthesis of silver nanoparticles.

### 2.3 Collection of silver nanoparticles

The silver nanoparticle solution was centrifuged for 20 min at 10,000 rpm (centrifugation machine, Sigma 3K30). The resulting pellet was washed with distilled water to wash off impurities. The resulting solution was lyophilized (lyophilizer: Christ Alpha 1-4 LD) to get powdered shiny silver nanoparticles.

### 2.4 Optimization of various parameters for silver nanoparticle synthesis

The parameters (time, pH, temperature, concentration of silver nitrate, and plant extract) were optimized for the rapid and maximum synthesis of AgNPs. The pH was maintained at 1, 3, 5, 7, 9, and 11 and optimized with 0.1 N hydrogen chloride (HCl) and 0.1 N sodium hydroxide (NaOH). The reaction was monitored from 0 to 60 min for optimal production of AgNPs. The synthesis of silver nanoparticles was monitored at various incubation temperatures (25, 50, 75, and 100°C). The concentration of AgNO_3_ was optimized at different concentrations (0.50, 1.0, 1.5, and 2.0 mM). Similarly, the concentration ratio of plant extract and AgNO_3_ was optimized by increasing the concentration of plant extract in a 1 mM AgNO_3_ solution. The synthesis was observed at various plant extracts to silver nitrate solution ratios. The absorbance of nanoparticles was noted within the range of 300–800 nm by a UV–visible spectrophotometer.

### 2.5 Stability study

The optimized solutions of nanoparticles were kept in the dark for 30 days, and the stability of synthesized nanoparticles was determined by UV-Vis spectral analysis.

### 2.6 Characterization of green synthesized silver nanoparticles

#### 2.6.1 Visual inspection

The reduction of metal ions was visually inspected for color change in the reaction medium of AgNPs.

#### 2.6.2 UV-Vis spectroscopy

The wavelength of maximum absorbance (ʎmax) was scanned through UV-Vis spectra at the range of 300–800 nm with a spectrophotometer (UV 4000).

#### 2.6.3 Zeta analyzer

Zeta potential and size of silver nanoparticles were detected with Zetasizer Nanoseries (Malvern instrument).

#### 2.6.4 Scanning electron microscopy

Morphology and size of AgNPs were determined by a scanning electron microscope (Quanta 250 FEG) operating in the high-vacuum mode with an acceleration voltage of 15 kV.

#### 2.6.5 FTIR analysis

The FTIR spectrum of silver nanoparticles and plants was obtained by mixing isolated AgNPs and plants with potassium bromide (KBr) pellet. The resulting mixture was analyzed by an FTIR (Shimadzu) instrument.

### 2.7 Antimicrobial activity

The comparative antibacterial activity of the plant extracts, AgNPs synthesized from respective extracts, and commercially available antibiotics (standard: Ramficin) were evaluated against Gram-positive (*B. subtilis* and *S. aureus*) and Gram-negative (*Pasteurella multocida* and *E. coli*) bacterial strains through the agar-well diffusion method (Aziz et al., 2014). Ramficin was used as the positive control, while distilled water was used as the negative control. The antimicrobial and cytotoxicity assays were performed in the Bioassay Section, Medicinal Biochemistry Lab of the University of Agriculture, Faisalabad, from where bacterial strains were procured.

### 2.8 Cytotoxicity study by hemolysis assay

Cytotoxic effect was studied by performing a hemolysis test (Sadhasivam and Durairaj, 2014). For this assay, Triton X-100 (0.1%) was used as the positive control and phosphate-buffered saline (PBS) was used as the negative control. Blood sample (10 ml) was taken out of which 4 ml was added with 8 ml of 0.2M D-PBS (pH 7). The mixture was centrifuged at 10,000 rpm for 10 min. The pellet containing red blood cells (RBC_s_) was further washed by D-PBS at 10,000 rpm. The RBCs obtained were diluted with 40 ml D-PBS. From this test sample, positive and negative controls were prepared. The samples were kept for incubation at room temperature for 4 h. It was vortexed and centrifuged at 10,000 rpm for 3 min. The supernatant was collected, and the optical density (OD) value was observed at 576 nm using an enzyme-linked immunosorbent (ELISA) plate reader. Percentage hemolysis was calculated as follows:

%Hemolysis=  Mean OD of sample−Mean OD of negative control  Mean OD of positive control− Mean OD of negative control × 100.

### 2.9 Statistical analysis

The results were expressed as mean ± SEM and analyzed by one-way ANOVA followed by a t-test in SPSS v. 21.

## 3 Results and discussion

### 3.1 Green synthesis of silver nanoparticles

The visible color change from light yellow to brownish yellow indicated fast formation and nucleation of AgNPs. The appearance of brown color could be explained due to the surface plasmon vibration effect and change of Ag^+^ ions to Ag^o^ by aqueous extracts. The antibacterial activity of *E. camaldulensis* extract has been due to the components which were indicated as gallic acid, taxifolin, methyl gallate, quercetin, luteolin, and hesperidin (Ghareeb et al., 2018). Considering the therapeutic importance of gallic acid, we assume it as a reducing agent. Similarly, arjunic acid, arjungenin, arjunetin, and luteolin are antimicrobial components in *T. arjuna* (Aneja et al., 2012). Due to the close resemblance of luteolin with quercetin which has known value in nanoparticle synthesis, we supposed it to be responsible for the bio-reduction of silver metal ions.

$$AgNO_{3}\to Ag^{+}+NO_{3^{-}},$$ (1)

$$Ag^{+}+flvonoids\;/\;polyphenolic\;-\text{OH},\text{ CHO},\text{ etc}.=Ag^{0}\text{ nanoparticles}\text{.}$$ (2)

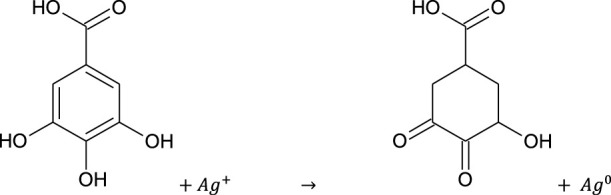



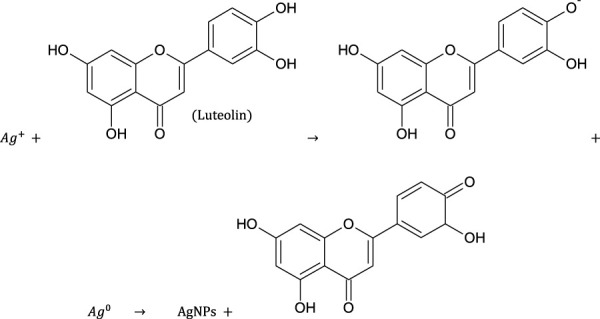


### 3.2 Visual/UV-Vis spectral analysis

**FIGURE 1:**
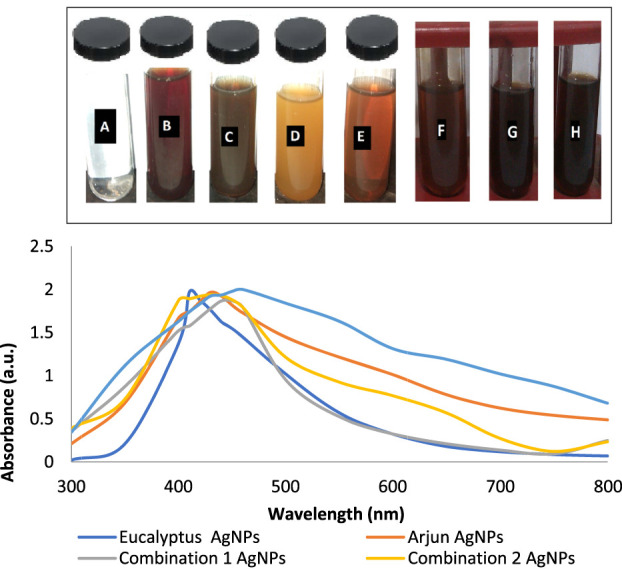
**(A)** Visual inspection of color change as silver nanoparticles synthesized **(**A: aqueous AgNO_3_ solution, B: *T. arjuna* extract, C: AgNPs with *T. arjuna*, D: *E. camaldulensis* extract, E: AgNPs with *E. camaldulensis*, and F–H: AgNPs from combinations 1–3); **(B)** UV–visible spectra of green synthesized silver nanoparticles.

The color of *E. camaldulensis* extract was changed from light yellow to orange-brown, while the reddish-brown color of *T. arjuna* was turned to dark brown by adding an aqueous solution of AgNO_3_. Similarly, the change in color was observed when extract combinations were added to the aqueous solution of AgNO_3_ as shown in Figure 1A. Synthesis of metallic AgNPs was further confirmed with the UV-Vis characteristic absorption band of silver nanoparticles at a wavelength of 300–800 nm (Figure 1B). The surface plasmon resonance band showed maximum absorbance peaks of AgNPs at 410, 430, 440, 420, and 460 nm when synthesized with individual extracts of *E. camaldulensis*, *T. arjuna*, and their combinations 1, 2, and 3, respectively. The surface plasmon resonance bands are influenced by the shape, morphology, size, dielectric environment, and composition of the synthesized NPs (Shameli et al., 2012). According to Mie’s theory, spherical metal NPs can only give a single SPR band, while anisotropic particles could give two or more than two SPR bands depending upon the shape of NPs (He et al., 2002). In the present study, a single SPR peak was observed which proposed that synthesized silver nanoparticles were spherical in shape.

### 3.3 Optimization of conditions for synthesis of silver nanoparticles

**FIGURE 2:**
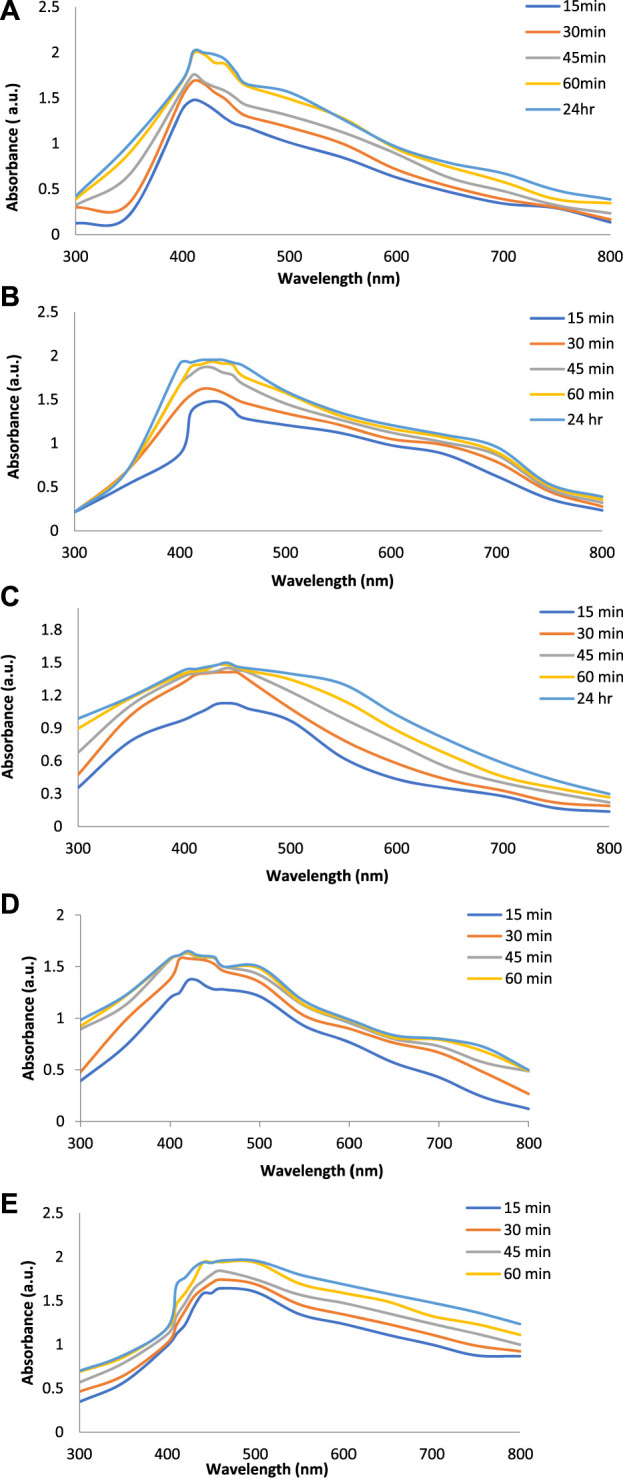
Effect of time on synthesis of AgNPs from **(A)** *E. camaldulensis*, **(B)** *T. arjuna*, **(C)** combination 1, **(D)** combination 2, and **(E)** combination 3.

**FIGURE 3:**
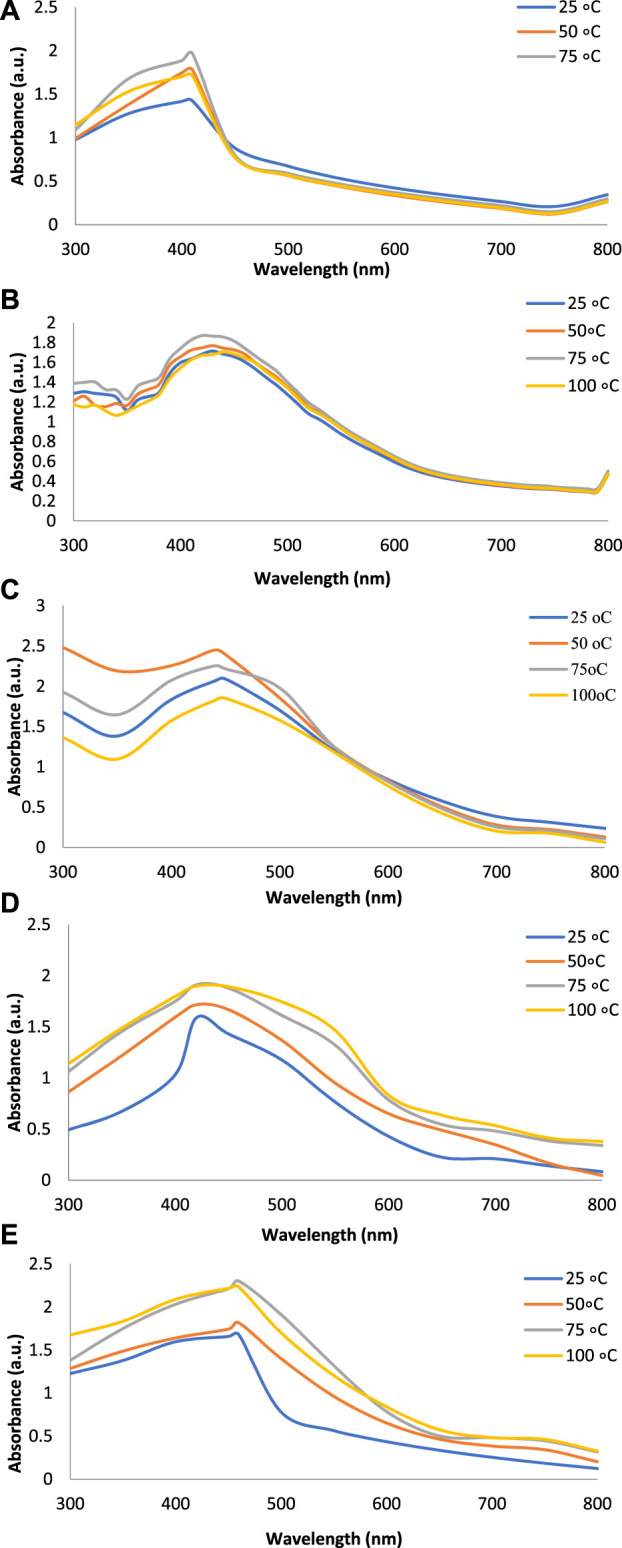
Effect of temperature on synthesis of AgNPs from **(A)** *E. camaldulensis*, **(B)** *T. arjuna*, **(C)** combination 1, **(D)** combination 2, and **(E)** combination 3.

**FIGURE 4:**
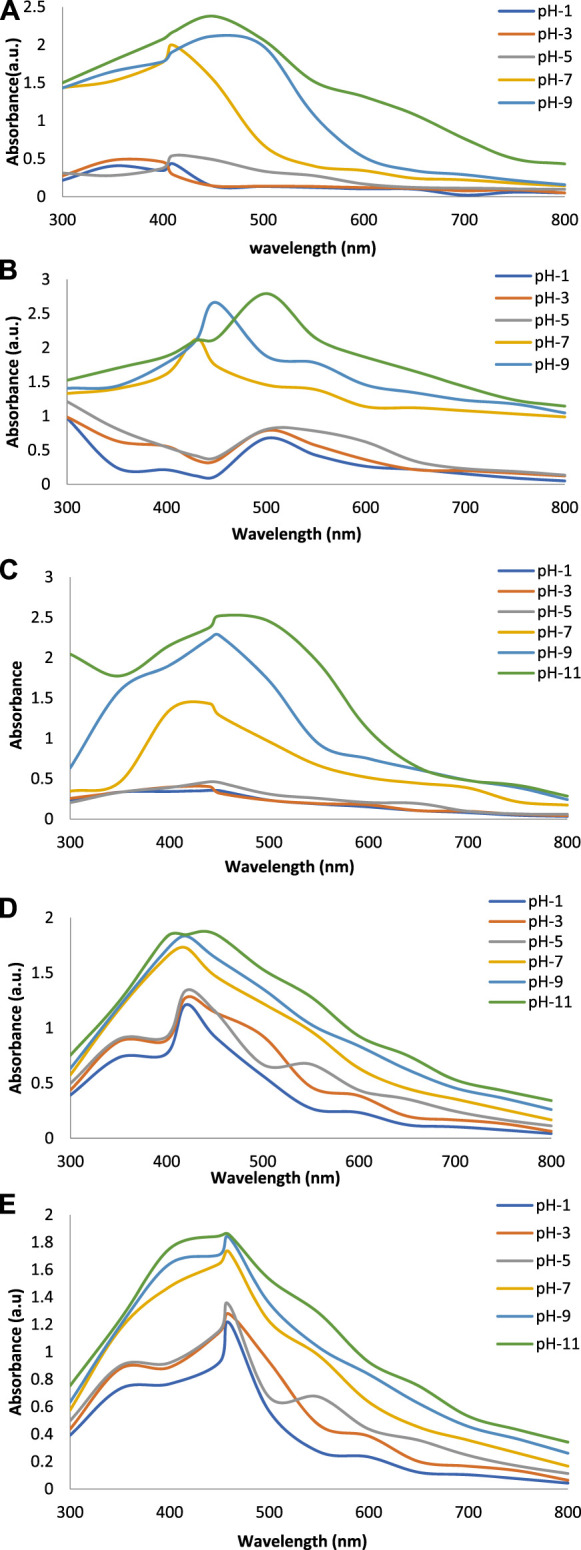
Effect of pH on synthesis of AgNPs from **(A)** *E. camaldulensis*, **(B)** *T. arjuna*, **(C)** combination 1, **(D)** combination 2, and **(E)** combination 3.

**FIGURE 5:**
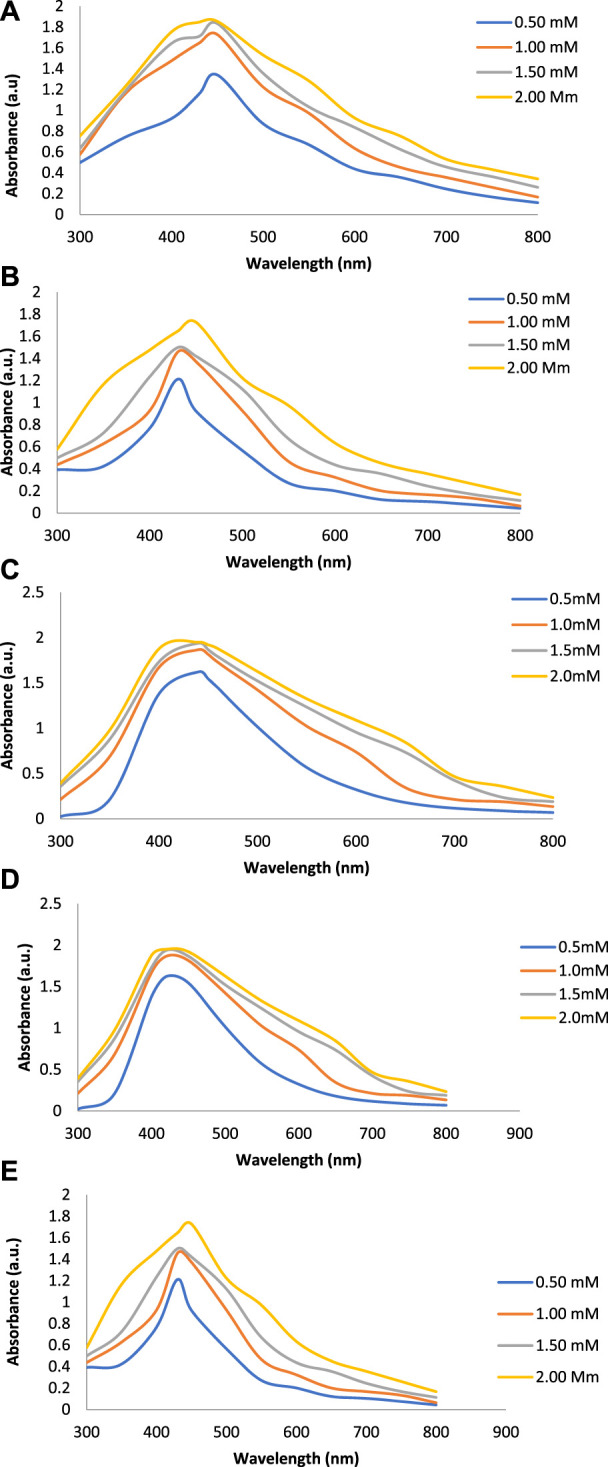
Effect of AgNO_3_ concentration on synthesis of AgNPs from**(A)** *E. camaldulensis*, **(B)** *T. arjuna,* **(C)** combination 1, **(D)** combination 2, and **(E)** combination 3.

**FIGURE 6:**
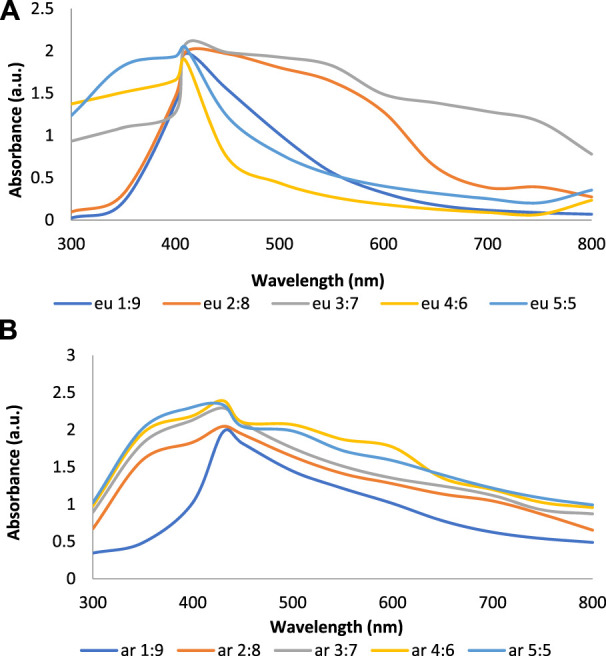
Effect of **(A)** *E. camaldulensis* **(B)** *T. arjuna* concentration on AgNPs synthesis.

Figures 2A–E show the UV-Vis spectra of AgNPs synthesize at various reaction times (15, 30, 45, and 60 min) with extracts of *T. arjuna*, *E. camaldulensis,* and their combinations. By increasing the reaction time from 15 to 60 min, the intensity of the absorption peak was increased. The optimum incubation time for synthesis was 60 min. Spectra revealed that SPR peaks of AgNPs with high absorbance were at 410–430 nm when synthesized with extracts of both plants, which is very speciﬁc for silver nanoparticles (Figures 2A, B). The UV-Vis spectra recorded after 24 h of the reaction showed no change in absorbance at specific ʎmax, conﬁrming that the optimum reaction time was 60 min. The incubation time was reduced from 60 to 30 min when combinations were used as reducing and stabilizing agents (Figures 2C–E). Instead of a single plant extract, plants in combination synthesized nanoparticles in a shorter time with better characteristics. In combinations, the bioactive components certainly imparted a synergistic effect for bio-reduction of Ag^+^ ion into Ag^0^ nanoparticles. The rise in temperature from 25°C to 75°C resulted in an increase in the absorbance, indicating an increased synthesis rate of AgNPs. The optimum temperature for the synthesis of silver nanoparticles was noted as 75°C because maximum absorbance at characteristic ʎmax of AgNPs was noted. Further increase in temperature not only decreased the absorbance but also shifted the λmax (Figures 3A–E). High temperatures (>100°C) produced larger particles. The better nano-sizing at low temperatures is possibly due to a reduction in the aggregation of growing AgNPs (Ibrahim, 2015). The UV-Vis spectra revealed that acidic media (pH 1–5) were not favorable for the synthesis of AgNPs. Moreover, the acidic medium lightened the color of the silver nanoparticle solution. The size of nanoparticles is directly influenced by pH. In acidic media compared to a basic one, the particle size is anticipated to be greater as well (Khalil et al., 2014). These pH influences in the size-effect can be seen in the colors, which range from colorless to yellow (Gontijo et al., 2020). The ability of the reaction pH to alter the electrical charges of biomolecules, which may have an impact on their capacity for capping and stabilizing, as well as the subsequent growth of the nanoparticles, is one of its significant influences. Prompt color change was observed in alkaline media (pH 9 and 11), but the shifting of characteristic peaks toward longer wavelengths in spectra did not support the formation of nanoparticles. Moreover, agglomeration of nanoparticles was also observed at pH 11 (Figures 4A–E). The pH 7, with 60 min of incubation with all green treatments, showed maximum absorbance at characteristic surface plasmon resonance (SPR) peaks. Therefore, the neutral pH (7) is recommended as the optimum pH. Absorbance spectra showed that the low concentration (0.5 mM) of AgNO_3_ was found to be insufficient for the formation of nanoparticles. Maximum synthesis of nanoparticles was found at a 1 mM concentration. However, a 1.5 mM solution showed almost the same results. The shifting of the SPR peaks of AgNPs at a high concentration of AgNO_3_ (2 mM) indicated this was unsuitable for the synthesis of AgNPs. Therefore, the optimum concentration of AgNO_3_ was 1 mM (Figures 5A–E) in agreement with Krishnaraj et al. (2012). The absorption spectra showed that the most appropriate ratio of *E. camaldulensis* extract and silver nitrate solution was 3:7 for maximum production of silver nanoparticles, while *T. arjuna* gave a better yield with the ratio of 4:6 (Figure 6A, B).

### 3.4 Stability study

The stability of AgNPs was studied by UV-Vis spectroscopy after 30 days. Green synthesized AgNPs showed a characteristic SPR peak at the same ʎmax with the same absorption intensity. This clearly indicated the strong stability of AgNPs for 30 days, which was very supportive and convenient for the synthesis of nanoparticles.

### 3.5 Characterizations of silver nanoparticles

#### 3.5.1 Zeta potential

**FIGURE 7:**
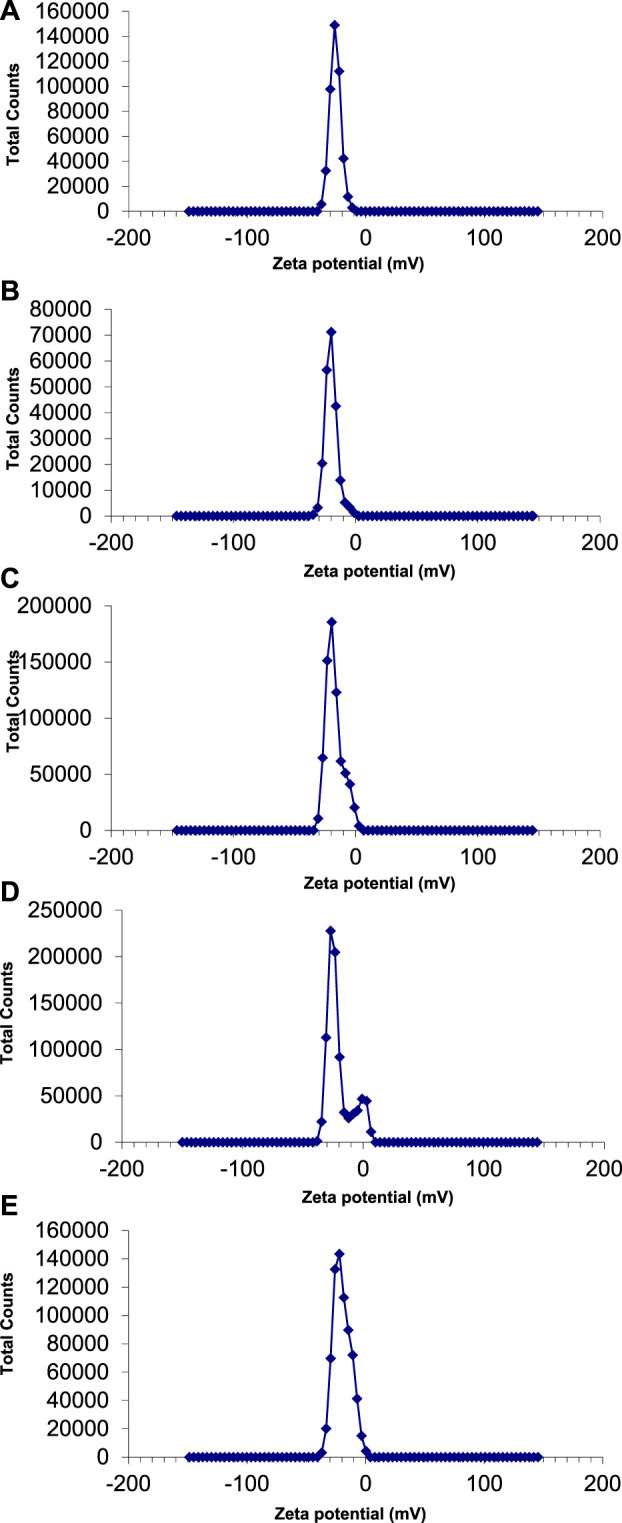
Zeta potential of AgNPs synthesize with **(A)** Eucalyptus **(B)** *T. arjuna* **(C)** combination 1 **(D)** combination 2 **(E)** combination 3.

The zeta potential values of AgNPs synthesized with *E. camaldulensis* and *T. arjuna* were -26 mV ± 4.61 mV and -20 mV ± 5.09 mV, respectively (Figures 7A, B). Zeta potential measurement of both *E. camaldulensis* and *T. arjuna* treated AgNPs gave a single peak with peak to area ratio of 25.9 mV/100.0% and 20 mV/100.0%, respectively. In the case of AgNPs synthesized in the presence of combinations 1, 2, and 3, zeta potential values were −20 mV ± 10.3 mV, −27 mV ± 6.9 mV, and −22 mV ± 7.1 mV, respectively, as shown in Figures 7C–E. A minimum ± 30 mV value of zeta potential is required for stable nanosuspension (Aruna et al., 2014). The negative zeta potential value depicted by the silver nanoparticles may be due to the potential capping of the bioorganic components present in the plant’s extracts (Edison and Sethuraman, 2012). The high negative values reveal the electrostatic repulsion between the particles and facilitate the achievement of stable silver nanoparticles without any agglomeration (Sivaraman et al., 2013). It was concluded that AgNPs prepared with combination 2 have a significantly high zeta potential value (−27 mV), indicating more electrostatic repulsion between the particles and ultimately high stability.

#### 3.5.2 Dynamic light scattering

**FIGURE 8:**
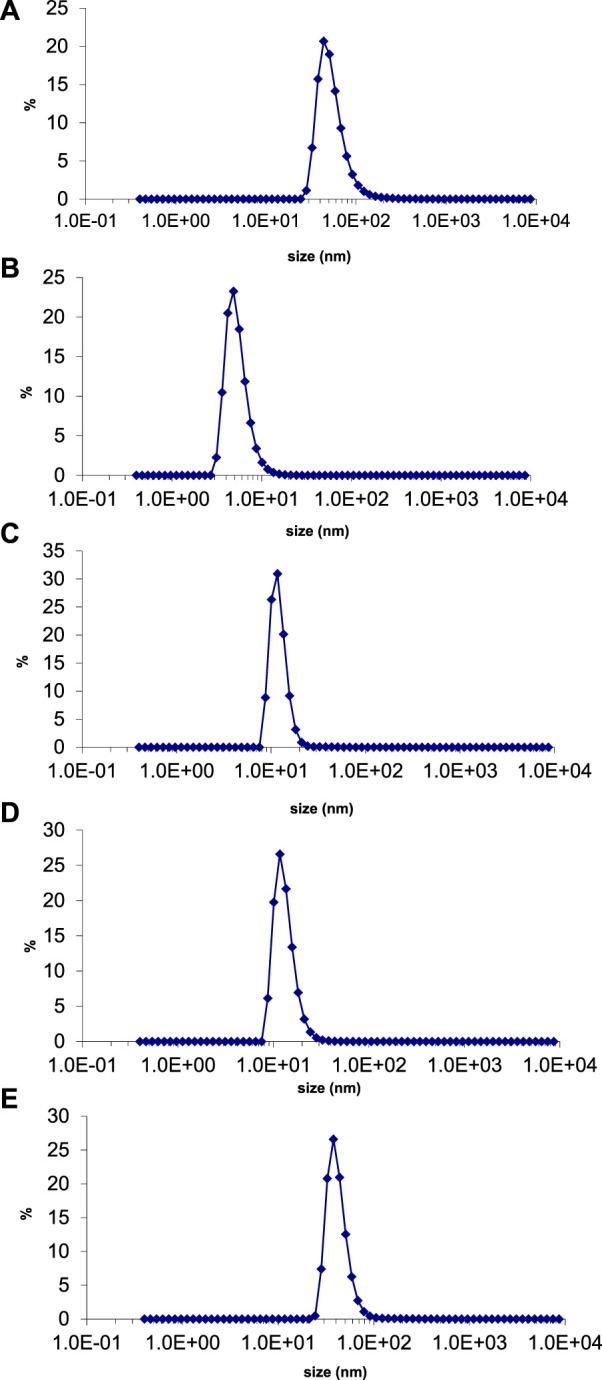
Particle size distribution of AgNPs synthesized from **(B)** *T. arjuna* extract **(B)** *T. arjuna* extract **(C)** Combination 1 **(D)** Combination 2 **(E)** Combination 3.

The average particle size and polydispersity index (PDI) of green synthesized silver nanoparticles were evaluated by using a nano zetasizer. The theory behind the size determination of nanoparticles in solution is dynamic light scattering (DLS). It determines the diameter of the nanoparticle by light scattering properties and Brownian motion of particles (De Assis et al., 2008). The average size of silver nanoparticles synthesized with *E. camaldulensis*, *T. arjuna,* and combinations 1, 2, and 3 was 43, 23, 12, 13, and 42 nm, respectively (Figures 8A–E). PDI of silver nanoparticles manufactured with *E. camaldulensis* and *T. arjuna* and combinations 1, 2, and 3 was 0.399, 0.716, 0.478, 0.683, and 0.433, respectively. The PDI value greater than 0.7 indicates the polydisperse nature of particles, while less than 0.7 indicates monodispersity (Honary et al., 2013). Monodispersed silver nanoparticles showed higher performances and unique applications as compared to the polydisperse nanoparticles. Results showed that silver nanoparticles synthesized with *T. arjuna* and combinations 1, 2, and 3 were monodispersed, while nanoparticles synthesized with *E. camaldulensis* were polydispersed.

#### 3.5.3 Scanning electron microscopy

The SEM micrographs showed that spherical and uniform AgNPs were synthesized with a diameter of 100 and 35 nm in the presence of *E. camaldulensis* and *T. arjuna*, respectively. Similarly, silver nanoparticles fabricated in combinations 1, 2, and 3 attained the sizes 37, 46, and 80 nm, respectively.

#### 3.5.4 FTIR studies

The potential biomolecules responsible for the reduction of Ag^+^ into Ag^0^ NPs were identified by FTIR spectroscopy. The FTIR spectrum of *E. camaldulensis* indicated absorption peaks at 3658.96, 3319.49, 2830.79, and 1703.14 cm^−1^, representing characteristic stretching bands of O–H, N-H, C-H, and C=O groups, respectively. The characteristic absorption peaks due to bending vibrations of aromatics, amines, amides, and acids were between 900 and 650 cm^−1^. The silver nanoparticles synthesize from *E. camaldulensis* showed absorption peaks of OH (3653.16 cm^−1^), N-H (3317.56 cm^−1^), C=O (1701 cm^−1^), and aromatic (908.47 cm^−1^) functional groups. The peaks situated at 3651.25, 3338.78–3240.41, 1701.22, and 1695.50 cm^−1^ in spectra of *T. arjuna* aqueous extract are characteristic peaks for O–H, N–H, C–H, C=O, and amide stretching, respectively. The AgNPs synthesized from *T. arjuna* extract showed peaks due to stretching vibration at 3649.32 cm^−1^ (O-H stretch), 3336.65–3238.46 cm^−1^ (N-H stretch), and 1693.50 cm^−1^ (C=O stretch). The peaks located at 908 cm^−1^ and onward are due to bending vibrations of the groups. The similarities between peaks in spectra of plant extracts and nanoparticles synthesized in plant extract with some nominal shifts confirmed the presence of plant extract in nanoparticles as capping and stabilizing agents. In the FTIR spectrum, the appearance of peaks in hydroxyl, carbonyl, C=C, amide, and amine group regions is characteristic of polyphenols, alkaloids, flavonoids, fatty acids, amino acids, and enzymes/proteins (Krishnaraj et al., 2012). It has been reported that extracts of *E. camaldulensis* have tannins, alkaloids, and saponins (Sani et al., 2014). Similarly, *T. arjuna* extract is a rich source of polyphenols, flavonoids, tannins, and glycosides (Mandal et al., 2013). The shifting of the C=O peak in spectra obtained from silver nanoparticles indicated that carbonyl-containing functional groups may be responsible for bio-reduction of silver ions into silver nanoparticles while shifting of stretching vibrations of O-H and N-H to lower wavelengths indicated binding of these functional groups with the surface of silver nanoparticles (Shafaghat, 2015).

### 3.6 Antimicrobial and hemolytic activity

#### 3.6.1 Antibacterial assay

AgNPs synthesized with *E. camaldulensis*, *T. arjuna*, and combinations 1, 2, and 3 showed a significantly high zone of inhibition against Gram-positive strain than to Gram-negative strains. Silver nanoparticles synthesized with combination 2 and *T. arjuna* showed the highest zone of inhibition (16 mm) against *B. subtilis*, while combination 3 showed the largest zone of inhibition against *S. aureus* (Table 1). The antibacterial potential of AgNPs was comparable to that of the standard drug Ramficin. The antimicrobial effects of AgNPs against multidrug-resistant bacteria have been studied by researchers, and it was proved that AgNPs are effective against multidrug-resistant bacteria. Antibacterial activities of silver nanoparticles prepared from *Gongronema latifolium* extract (Aisida et al., 2019a), *Veronica amygdalina* extract (Aisida et al., 2019b), and other medicinal plants have been reported previously. However, the inhibition zones from the studies showed the range through a large extent of variation. Therefore, the comparison of the results is difficult as there is no standard method for the determination of the antibacterial activity of AgNPs and different methods have been applied by the researchers. Shameli et al. (2012) have presented bactericidal potencies (https://www.ncbi.nlm.nih.gov/pmc/articles/PMC7973307/table/tbl1/?report=objectonly) of nanoparticles. In this study, AgNPs exhibit good antibacterial activity, particularly against Gram-positive bacteria. As suggested by Loo et al. (2018), less susceptibility of Gram-negative bacteria may be due to the positive charges of AgNPs trapped and blocked by lipopolysaccharide, thus making them less susceptible. The exact mechanisms of AgNPs against bacteria still remain unknown. However, there are some proposed mechanisms that the action of AgNPs on bacteria may be due to its ability to interact with membrane phospholipids, rupturing of the cell membrane, physical interference with cellular components, ROS generation, interaction with cytosolic proteins and enzymes, and elevated metal ion concentration (Hameed et al., 2019). The size and morphology of nanoparticles actually determine the toxicity, biological fate, *in vivo* distribution, and targeting potential (Pal et al., 2011). The antibacterial potential of silver nanoparticles is due to membrane disruption of microbes with adhesive substances like proteins and polysaccharides and the bactericidal action of Ag^+^ ion (Mohammed 2015).

**TABLE 1:** Antibacterial potential of green synthesized silver nanoparticles.

	Zone of inhibition (mm)			
	*B. subtilis*	*S. aureus*	*E. coli*	*P. multocida*
EuC AgNPs	13.0 ±0.4*	13.0 ± 0.3*	8.0 ± 0.6	7.0 ± 0.1
TAr AgNPs	16.0 ± 0.09*^,#^	11.0 ± 0.5*	7.0 ± 0.7	6.0 ± 0.3
C_1_ AgNPs	16.0 ± 0.4*^,#^	14.0 ± 0.1*	11.0 ± 0.9	10.0 ± 1.3
C_2_ AgNPs	10.0 ± 0.6	15 ± 1.2*	12 ± 0.08	12 ± 0.5
C_3_ AgNPs	14 ± 0.5*	17 ± 0.4*^,#^	12 ± 0.4	10 ± 0.2
Ramficin (positive standard)	20 ± 0.4	17 ± 0.4	18 ± 0.5	18 ± 0.4

C_1_, combination 1; C_2_, combination 2; C_3_, combination 3; EuC, *E. camaldulensis*; TAr, *T. arjuna*.

^*^significantly different from Gram-negative bacteria (*p* < 0.05).

^#^significantly show the highest zone of inhibition (*p* < 0.05).

#### 3.6.2 Hemolytic activity

**FIGURE 9:**
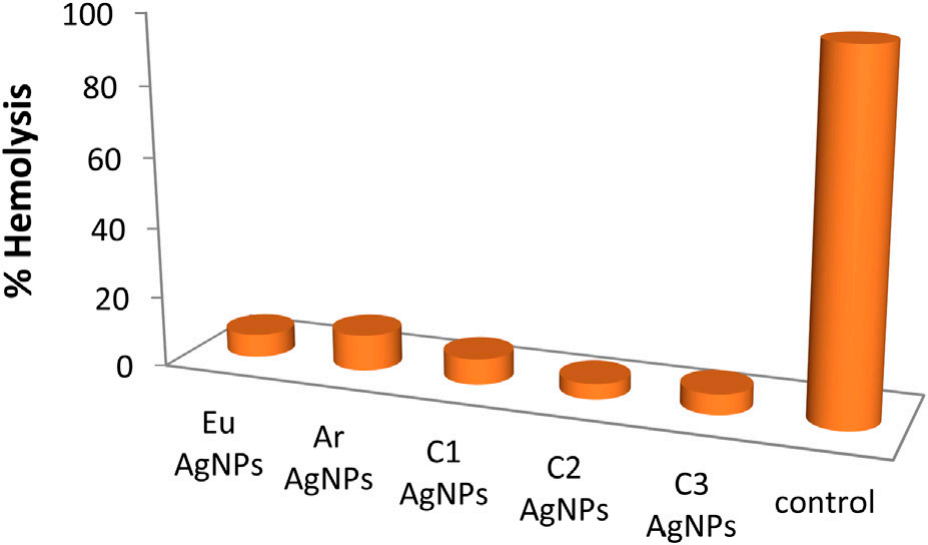
Hemolysis (%) caused by green synthesized silver nanoparticles.

AgNPs synthesized with combination 2 showed the least hemolytic activity (Figure 9), while AgNPs synthesized with *T. arjuna* caused relatively more hemolysis to red blood cells (RBCs). Triton-X showed 100% hemolysis. Overall, nanoparticles synthesized with plant extract were least toxic to RBCs than positive control Triton-X. For future work, silver metal oxide nanomaterials such as previously synthesized zinc iron oxide (ZnFe2O_4_) nanocomposite (Matinise et al., 2018), titanium oxide heterojunction (Sm_2_Ti_2_O_7_) photocatalyst (Kaviyarasu et al., 2020), iron oxide nanoparticles (Aisida et al., 2020), CuO nano-platelets (Sone et al., 2020), and bunsenite NiO nanoparticles (Sone et al., 2016) could be formulated and tested for biological activities (particularly antimicrobial activity based on this study) to compare the efficiency. It is further suggested to identify bioactive molecules in the natural extracts and elucidate the reaction mechanisms that govern the interaction of the bioactive molecules with the precursor Ag salt.

We report the successful synthesis of silver nanoparticles using an aqueous extract from leaves of *E. camaldulensis* and bark of *T. arjuna* and their combinations in ratios of 1:1 (combination 1), 2:1 (combination 2), and 1:2 (combination 3) by extracts of *E. camaldulensis* and *T. arjuna* as an effective biosynthesis agent. The antibacterial potential of these AgNPs was comparable to that of the standard drug Ramficin.

## 4 Conclusion

In the present study, aqueous extracts of *E. camaldulensis* and *T. arjuna* (individually and in combinations) were used as green-reducing and stabilizing agents for the synthesis of silver nanoparticles. It was indicated that the method adopted under optimized conditions of pH (7), temperature (75°C), time (60 min), and concentration of AgNO_3_ of 1 mM is the most appropriate method and capable of synthesizing stable and uniform-sized silver nanoparticles. Structural and optical characterization of these nanoparticles confirmed the formation of pure, spherical silver nanoparticles with an average particle size of 43, 23, 12, 13, and 42 nm by *E. camaldulensis*, *T. arjuna* extracts, and combinations 1, 2, and 3, respectively. AgNPs synthesized with combination 2 showed the highest zone of inhibition against *B. subtilis*, while combination 3 showed the largest zone of inhibition against *S. aureus*, showing the least hemolytic activity. In this plant-mediated synthesis, the combination of plant extracts imparted synergetic reducing and stabilizing potential and synthesized more stable nanoparticles as compared to the individual plants. The results showed that silver nanoparticles synthesized by the combination of the two aqueous extracts are considered promising candidates for the development of antibacterial agents.

## Data Availability

The original contributions presented in the study are included in the article/Supplementary Material;further inquiries can be directed to the corresponding author.

## Supplementary material

Supplementary Figure S1 SEM micrograph of silver nanoparticles synthesized from **(A)** *E. camaldulensis* extract, **(B)** *T. arjuna* extract, **(C)** combination 1, **(D)** combination 2, and **(E)** combination 3.

Supplementary Figure S2 FTIR spectrum of **(A)** *E. camaldulensis*, **(B)** AgNPs from *E. camaldulensis*, **(C)** *T. arjuna*, and **(D)** AgNPs from *T. arjuna*.
