# Supplementary material for: Green synthesized silver nanoparticles: Optimization, characterization, antimicrobial activity, and cytotoxicity study by hemolysis assay
Source: Front Chem. 2022 Aug 29;10:952006. doi: 10.3389/fchem.2022.952006 (PMC9465387; doi:10.3389/fchem.2022.952006)
Supplement: Supplementary file 1 [file DataSheet1.docx]

**a**
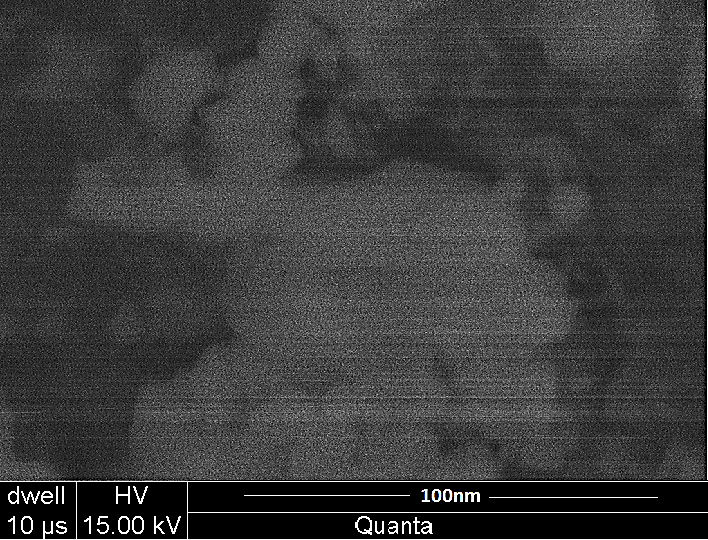


**b**
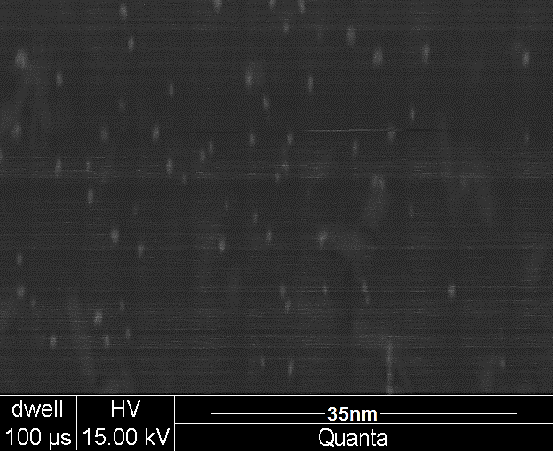


**c**
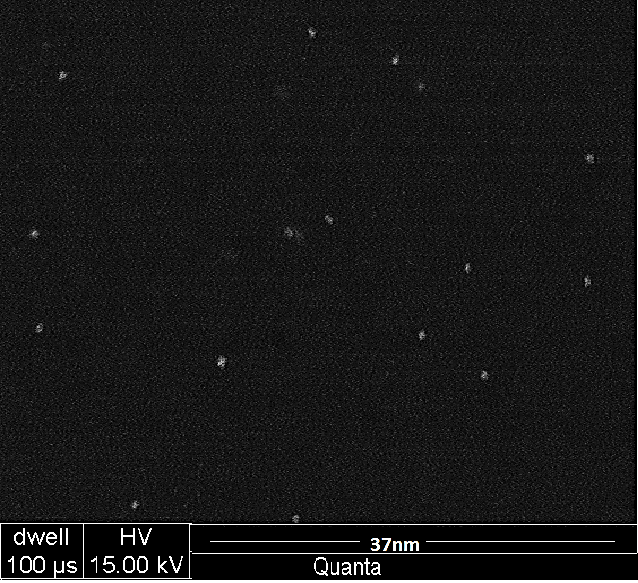


**d**
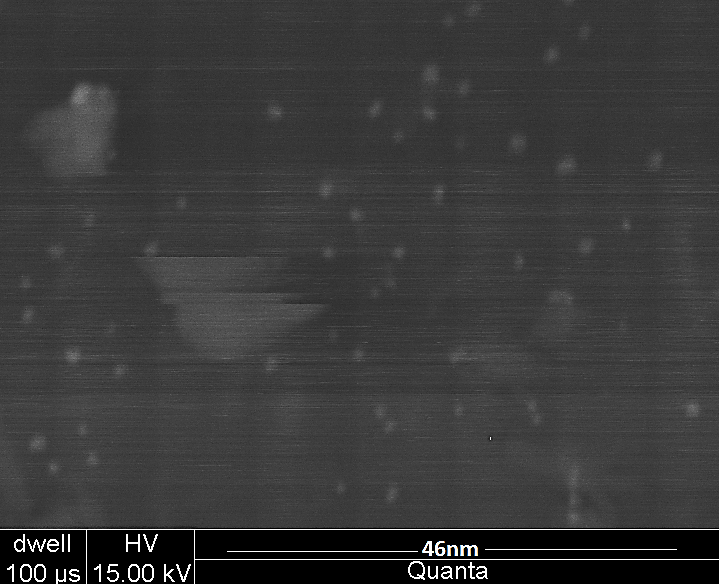


**e**
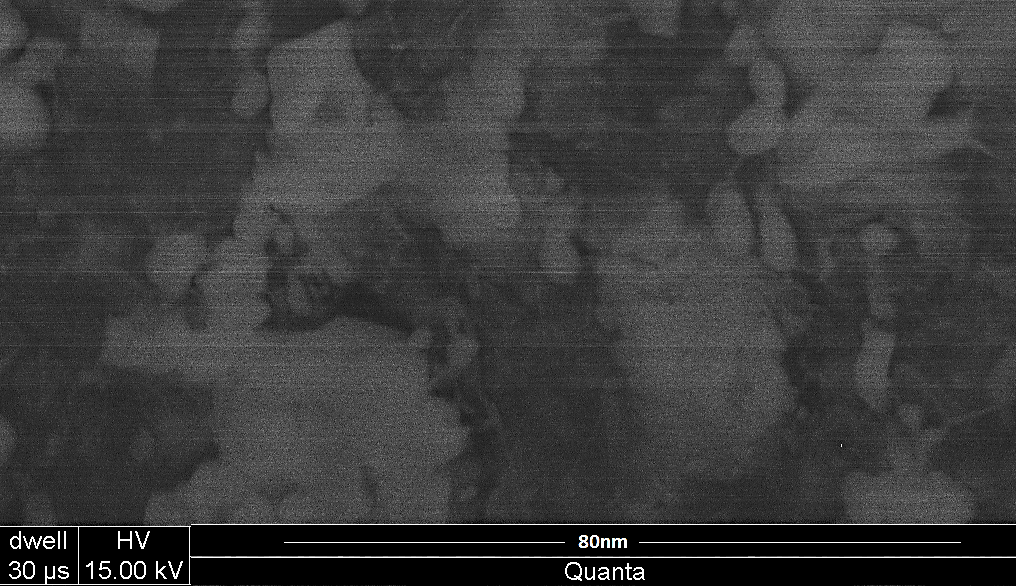


**Figure S1.** SEM micrograph of silver nanoparticles synthesize from (a) *E. camaldulensis* extract (b) *T. arjuna* extract (c) Combination 1 (d) Combination 2 (e) Combination 3

**
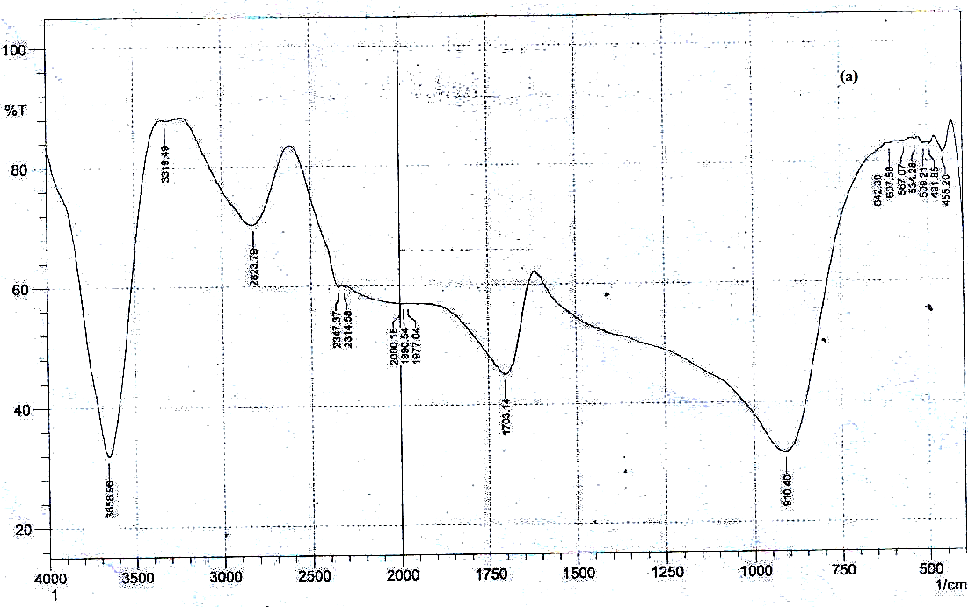
**

**
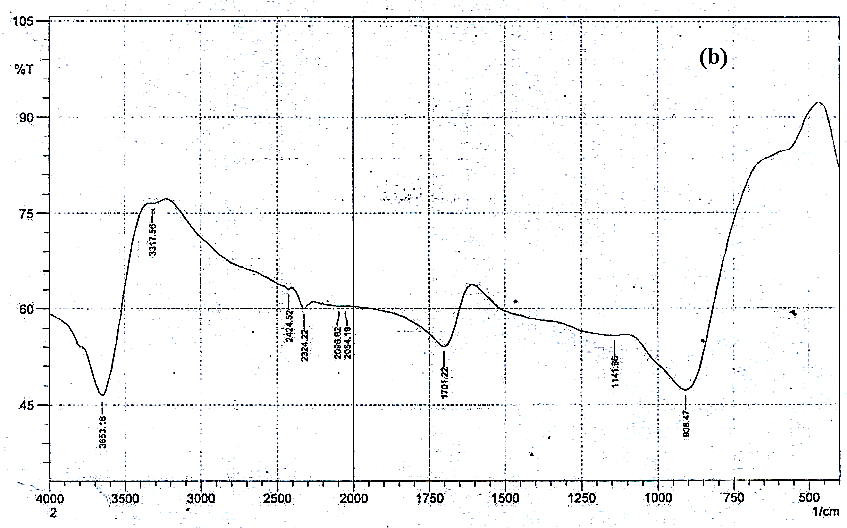
**

**
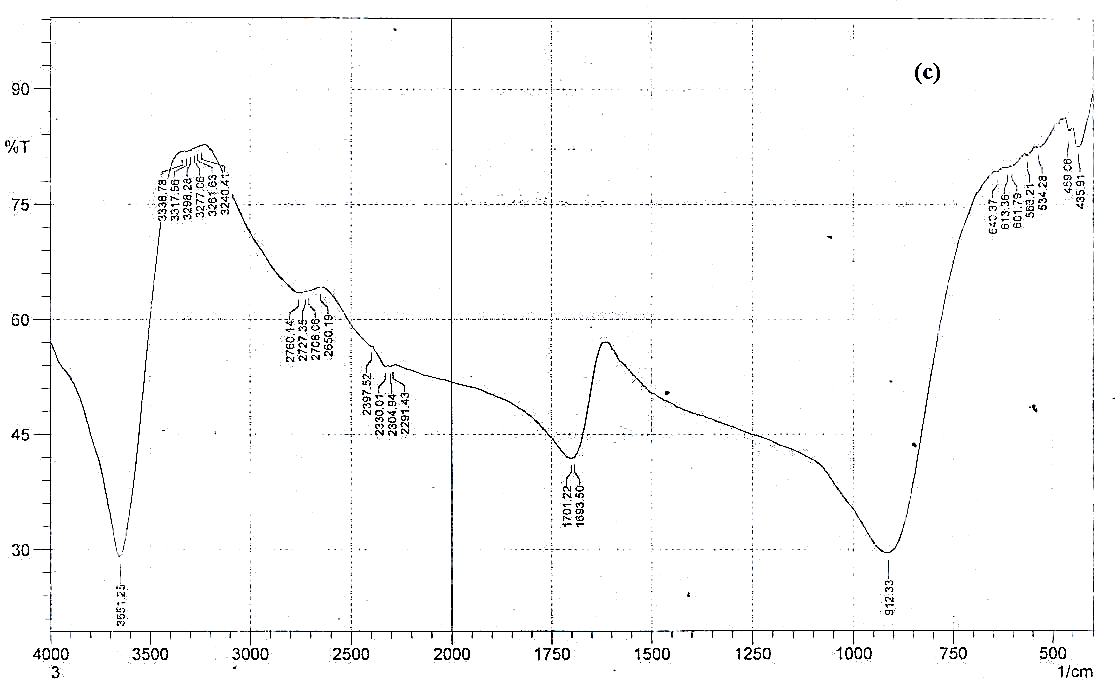
**

**
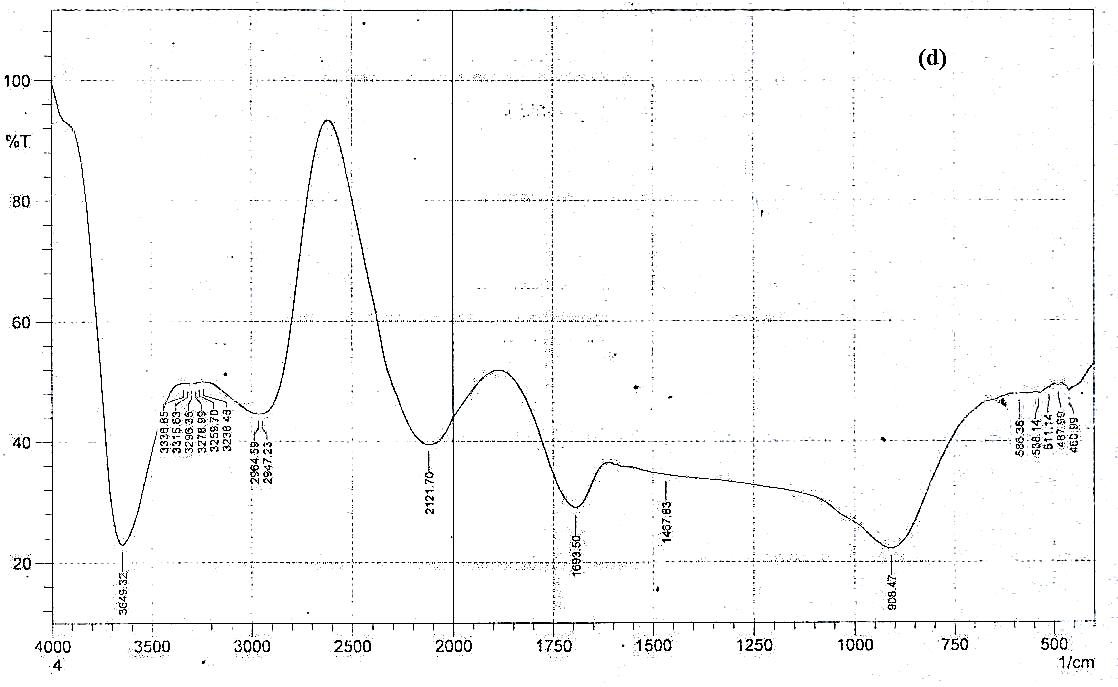
**

**Figure S2.** FTIR spectrum of (a) *E. camaldulensis* (b) AgNPs from *E. camaldulensis* (c) *T. arjuna* (d) AgNPs from *T. arjuna*
